# Resistance to age-related hearing loss in the echolocating big brown bat (*Eptesicus fuscus*)

**DOI:** 10.1098/rspb.2024.1560

**Published:** 2024-11-06

**Authors:** Grace Capshaw, Clarice A. Diebold, Danielle M. Adams, Jack G. Rayner, Gerald S. Wilkinson, Cynthia F. Moss, Amanda M. Lauer

**Affiliations:** ^1^ Department of Psychological and Brain Sciences, Johns Hopkins University, Baltimore, MD 21218, USA; ^2^ Department of Biology, University of Maryland, College Park, MD 20742, USA; ^3^ The Solomon H. Snyder Dept of Neuroscience, Johns Hopkins University School of Medicine, Baltimore, Maryland 21205, USA; ^4^ Department of Otolaryngology-HNS, Johns Hopkins University School of Medicine, Baltimore, MD 21205, USA

**Keywords:** presbycusis, auditory ageing, cochlea, Chiroptera

## Abstract

Hearing mediates many behaviours critical for survival in echolocating bats, including foraging and navigation. Although most mammals are susceptible to progressive age-related hearing loss, the evolution of biosonar, which requires the ability to hear low-intensity echoes from outgoing sonar signals, may have selected against the development of hearing deficits in bats. Many echolocating bats exhibit exceptional longevity and rely on acoustic behaviours for survival to old age; however, relatively little is known about the ageing bat auditory system. In this study, we used DNA methylation to estimate the ages of wild-caught big brown bats (*Eptesicus fuscus*) and measured hearing sensitivity in young and ageing bats using auditory brainstem responses (ABRs) and distortion product otoacoustic emissions (DPOAEs). We found no evidence for hearing deficits in bats up to 12.5 years of age, demonstrated by comparable thresholds and similar ABR and DPOAE amplitudes across age groups. We additionally found no significant histological evidence for cochlear ageing, with similar hair cell counts, afferent and efferent innervation patterns in young and ageing bats. Here, we demonstrate that big brown bats show minimal evidence for age-related hearing loss and therefore represent informative models for investigating mechanisms that may preserve hearing function over a long lifetime.

## 1. Introduction

Hearing is essential for echolocating bats that rely extensively on their auditory systems to forage, navigate and avoid obstacles. The evolution of echolocation in bats has been correlated with adaptations at all levels of auditory processing to enable active acoustic sensing of complex and dynamic environments. These include cochlear specializations to enhance the detection of behaviourally relevant frequencies (e.g. acoustic foveae in bats that use constant frequency echolocation calls [1,2]), and central processing specializations that facilitate the extraction of fine spectro-temporal cues from received echoes (reviewed in [3]). To receive detectable echo returns, bats emit intense sonar signals that can reach levels as high as 110–140 dB SPL (source levels re: 20 µPa at 0.1 m) [4–7]. Consequently, echolocating species frequently are exposed to intense self-generated sounds. Further, many species form high-density aggregations where sounds emitted by nearby conspecifics may be potentially damaging to the cochlea. The critical role of hearing in the fitness and survival of echolocating bats suggests that the evolution of this active sensing system may have introduced selective pressures to protect the auditory system from damage over a lifetime of exposure to sound.

The ageing auditory system in most mammals shows a progressive loss of hearing that begins with high-frequency deficits and extends to low frequencies over time [8,9]. Although the aetiology of age-related hearing loss (ARHL) is highly variable depending on genetic, epigenetic and environmental factors, its onset is generally correlated with senescent changes to peripheral auditory structures, including loss of inner and outer hair cells, loss of ribbon synapses and retraction of auditory nerve fibres (cochlear synaptopathy), and deterioration of the stria vascularis [8,10–13]. The molecular mechanisms underlying ARHL are hypothesized to result from inter-related metabolic and physiological changes over the lifespan that lead to the accumulation of reactive oxygen species and increase susceptibility to cellular dysfunction [14–16]. Recently, bats have emerged as a powerful model for ageing studies, due to their extended lifespans relative to comparably sized mammals [17–21]. Extreme longevity in bats is correlated with effective immune responses and reduced susceptibility to oxidative stress [22–25], leading to ‘healthy’ ageing characterized by reduced mitochondrial dysfunction and resistance to cellular senescence [26–28]. Bats, therefore, represent an informative model for understanding mechanisms that may support cochlear health over a long lifespan.

Echolocation requires the ability to hear quiet returning echoes within complex acoustic backgrounds. For bats that survive to old age, hearing deficits could lead to a multitude of negative outcomes, ranging from reduced foraging success to the inability to detect and avoid obstacles in flight. We hypothesize that echolocation imposes selective pressures to preserve hearing function across the lifespan, especially in species that require echolocation-based active sensing for prey capture. Although bats are not immune to hearing loss [29], and indeed, some species appear vulnerable to ARHL [30], recent comparative study has indicated that species differences in echolocation behaviours may correlate with differential susceptibility to hearing loss [31]. For example, non-echolocating bat species were highly susceptible to acoustic overexposure, with cochlear hair cell damage and loss comparable to that observed in mice, whereas echolocating bat species showed evidence for resistance to noise-induced hair cell damage [31]. Therefore, bats may vary in their susceptibility to cochlear ageing in a manner that correlates with their reliance on echolocation for survival. To date, ARHL has only been reported in the Egyptian fruit bat (*Rousettus aegyptiacus*) [30], a lingual echolocator that shows preference for visual over acoustic cues during behavioural tasks [32], and may not require acute auditory sensitivity across the lifespan compared to insectivorous echolocators that use their hearing to hunt prey.

In this study, we evaluated hearing sensitivity, outer hair cell function and cochlear morphology in young and ageing big brown bats (*Eptesicus fuscus*). *E. fuscus* is an insectivorous laryngeal echolocator that produces high-intensity calls (up to 138 dB SPL at 0.1 m [33]) to pursue and intercept prey in flight. This species has been reported to live up to 19 years in the wild [34,35] (mean longevity: 6 years [36]) and presumably good auditory sensitivity is essential for survival into old age, as foraging efficiency depends critically on the detection of weak, high-frequency echoes. Further, *E. fuscus* appears to be resistant to noise-induced hearing damage based on physiological and behavioural testing [37–39]. The specializations that confer resistance to noise damage in this species may enhance survival of cochlear hair cells and their afferent and efferent neurons into old age, which should preserve hearing sensitivity across the lifespan. Here, we predicted that young and ageing *E. fuscus* will have comparable peripheral auditory sensitivity to facilitate effective echolocation-based behaviours throughout their natural lifespan.

## 2. Methods

### (a) Animals

We assessed auditory sensitivity in 23 wild-caught *E. fuscus* collected in the state of Maryland (MD DNR permit #55440). Bats were housed with conspecifics with sufficient roosting locations, room to fly and access to food and water ad libitum. Colony housing was maintained at 21–27°C, with a relative humidity of 30–70%. All procedures were performed with the approval of the Johns Hopkins Animal Care and Use Committee and complied with the National Institutes of Health guide for the care and use of laboratory animals.

Bats were grouped by age into young (*n* = 13, eight female, five male) and ageing (*n* = 10, six female, four male) categories for analyses. Ageing phenotypes, including life stage definitions, have not been established in bats; therefore, we categorized bat age groups based on maximum and mean longevity estimates. Maximum longevity for *E. fuscus* is reportedly 19 years [35]; however, adult survivorship is low among wild populations, which show a 50% survivorship of 2–3 years [40–42], 20% survivorship past 6 years of age [35,40] and mean longevity of approximately 6.7 years [36]. In the present study, bats younger than 6.5 years of age were considered young and bats older than 6.5 years were considered to be ageing. We selected age groupings similar to those used in other mammalian ageing studies, where ‘old’ phenotypes show the onset of senescent physiological changes in some, but not all, biomarkers, and ‘very old’ phenotypes display more severe deficits in all ageing biomarkers [43]. For example, previous ageing studies using short-lived laboratory mice, comparably long-lived deer mice and Egyptian fruit bats have categorized animals as ‘old’ when age exceeds 40–50% maximum longevity [30,44–51].

### (b) Age estimation

The laboratory colony comprises wild-caught *E. fuscus* of known minimum ages based on collection dates. To allow for more precise age estimation, we used DNA methylation profiling following established procedures [52]. DNA was extracted from wing tissue biopsies (3–4 mm diameter) and processed using the Zymo Quick-DNA Miniprep Plus Kit (ZymoResearch, Orange, CA). Samples were submitted to the Clock Foundation (Torrance, CA) to measure the proportion of methylated and unmethylated sites using a custom Illumina microarray [53]. We estimated ages using a species-specific epigenetic clock generated using 59 *E. fuscus* of known age [52]. This DNA methylation procedure has been previously validated to provide estimates of chronological age with high accuracy (median absolute error of 0.265 years for *E. fuscus*) [52].

### (c) Auditory brainstem response recording

We recorded auditory brainstem responses (ABRs) following previously published procedures [29]. Briefly, bats were anesthetized via an intraperitoneal injection of 50 mg kg^–1^ ketamine and 30 mg kg^–1^ xylazine and placed on a 37°C warming pad in a sound-attenuating chamber lined with acoustic foam (IAC Acoustics). Evoked potentials were recorded using subdermal needle electrodes placed at the vertex of the skull (recording), along the mastoid (inverting) and in the shoulder (ground). ABRs were acquired using BioSigRZ software (Tucker Davis Technologies (TDT), Alachua, FL) at a 12 kHz sampling rate with a TDT Medusa 4Z preamplifier connected to a TDT RZ6 I/O processor.

ABRs were recorded in response to broadband clicks (0.1 ms) and tones from 4 to 84 kHz (5 ms tone pips with 0.5 ms cos^2^ gating). Acoustic stimuli were broadcast using free-field speakers (TDT MF1 speaker for clicks and frequencies < 60 kHz, TDT ES1 speaker for frequencies ≥ 60 kHz) placed 10 cm from the bat’s head and oriented to present sound along the longitudinal axis of the pinna. ABR stimuli were calibrated to 90 dB peak equivalent SPL using a one-quarter inch microphone (PCB Piezotronics, model 377C01). Stimuli were presented at a rate of 21 s^–1^ and were attenuated in 10 dB steps from 90 to 10 dB SPL. ABRs were averaged across 512 presentations and filtered from 0.3 to 3 kHz for analyses.

Detection thresholds were defined as the intermediate stimulus presentation level above which an evoked response was discriminable from noise and below which no response was observed, following published methods (e.g. [54–56]). Physiological recordings were collected prior to completion of age estimation procedures, and thresholds were determined using visual inspection by two independent, experienced observers blinded to age groups. ABR wave peak-to-trough amplitudes and latencies were extracted using an automated user-supervised software [57].

We assessed age effects on ABR thresholds and the amplitudes and latencies of individual ABR waves using linear mixed-effects models (LMMs) fit with restricted maximum-likelihood estimation. We compared thresholds across the fixed effects of stimulus frequency, age group, sex, and the interaction of frequency, age group and sex. We assessed age group differences in ABR wave amplitudes and latencies across the fixed effects of stimulus level, age group, sex, and the interactions of level, age group and sex. Wave amplitude data were log-transformed to meet assumptions of the LMMs. We removed non-significant interaction terms to reduce model complexity. For all LMMs, we included subject ID as a random effect to account for individual variation. We assessed the significance of fixed effects using a conditional *F*-test with Kenward–Roger’s correction for degrees of freedom. *Post hoc* analyses were performed using a Tukey honest significant difference correction for multiple comparisons. Because categorical age groupings could obscure age-related trends, we additionally assessed detection thresholds and ABR wave metrics as described above, with chronological age incorporated as a continuous fixed effect; however, results were unchanged and we therefore report analyses based on age groupings here and report results using chronological age in the electronic supplementary material (figure S2 and tables S2, S6, S7). We additionally evaluated the relationship between ABR wave amplitudes and latencies and chronological age as a continuous predictor using linear regression followed by hypothesis testing of the Pearson correlation coefficient (*r*) against the null hypothesis predicting no correlation (*r* = 0) of age and wave metrics. All statistical testing was performed in R v. 4.3.2 [58].

### (d) Distortion product otoacoustic emission recording

We assessed outer hair cell function in a subset of bats (young *n* = 3, ageing *n* = 4) using distortion product otoacoustic emissions (DPOAEs). DPOAEs were recorded using TDT BioSigRZ in anesthetized animals within a sound-attenuating chamber, as described in the ABR recording procedures above. Closed-field acoustic stimuli were two simultaneous, iso-intensity tones (*f*_1_ and *f*_2_, *f*_2_ frequency range: 8–32 kHz and *f*_1_ presented at an *f*_2_ */f*_1_ ratio of 1.2) presented by two speakers (TDT MF1) coupled to an ear insert that was tightly inserted into the ear canal. Speakers were calibrated in-ear for each subject to account for variability in ear canal characteristics. Stimuli were presented from 80 to 20 dB SPL in 10 dB decrements. DPOAEs (2*f*_1 _– *f*_2_) were recorded using a low-noise probe microphone (TDT DPM1, flat frequency response 3–40 kHz) coupled to the ear insert and were averaged over 512 presentations. We additionally recorded control DPOAEs in a deceased bat to confirm that the data reflected active physiological processes rather than distortions generated as stimulus artefacts. We extracted DPOAE amplitudes per stimulus level to generate input–output functions and compared these across age groups using LMMs in R, incorporating subject ID as a random effect. We replicated these analyses replacing age group with chronological age as a continuous fixed effect, as described above. The results of this linear regression were not different from the age group analyses and are reported in electronic supplementary material, table S9.

### (e) Cochlear immunolabelling

We performed immunohistochemistry to label whole-mount cochlear dissections in a subset of bats (young *n* = 7, ageing *n* = 4) for a quantitative assessment of cochlear structures across age groups. When possible, bats were transcardially perfused with 4% paraformaldehyde prior to harvesting cochleas; however, in some cases, cochleas were obtained post-mortem. Cochleas were post-fixed in 4% paraformaldehyde at 4°C for at least 24 h prior to decalcification using 0.5M EDTA. Cochleas were then placed into blocking buffer (5% normal goat serum, 10% bovine serum albumen and 0.5% Triton X-100 (Electron Microscopy Services)) for 1 h, followed by 24 h incubation in primary antibodies diluted in blocking buffer. Primary antibodies included anti-myosin 6 (Myo-6, 1 : 500, Bioss, bs-11264R) to label cochlear hair cells, anti-synaptic vesicle protein (SV2, 1:500, DSHB, RRID: AB_2315387) to label efferent olivocochlear (OC) synapses and anti-C-terminal binding protein (CtBP2, 1 : 200, BD Transduction Laboratories, 612044, RRID: AB_399431) to label presynaptic ribbons. Cochleas were transferred to secondary antibodies in blocking buffer for 2 h at 20°C. Secondary antibodies used were Alexa Fluor 488 goat anti-mouse SFX (Molecular Probes, A31619) and Alexa Fluor 568 goat anti-rabbit IgG (Invitrogen, A11036).

Cochleas were dissected into 4–5 flat turns, mounted on slides using DAPI-Fluoromount-G medium (Southern Biotech) and imaged at 10× using a Nikon Eclipse Ti2 confocal microscope. Images were exported and the Measure_Line plugin for ImageJ (Eaton-Peabody Laboratories, Massachusetts Eye and Ear) was used to identify eight equidistant locations along the cochlea based on percent total length. Confocal *z*-stacks (*z* = 0.15 µm) of these eight locations were generated using a 60×/1.4 NA oil-immersion lens. Cochlear structures were analysed using Fiji [59]. Inner and outer hair cells were quantified per 180 µm continuous segment of each region. Counts and surface area measurements of CtBP2-positive synaptic ribbon puncta and SV2-positive efferent terminals were automatically extracted using the Analyze Particles function in Fiji. We analysed age-related differences in cochlear structures using LMMs in R, including age group and cochlear location as fixed effects and subject ID as a random effect. We replicated these models replacing age group with chronological age as a continuous fixed effect as described above; however, the results were not different from the age group analyses and are reported in electronic supplementary material, table S10.

## 3. Results

### (a) Physiological hearing sensitivity does not differ across young and ageing bats

**Figure 1.**
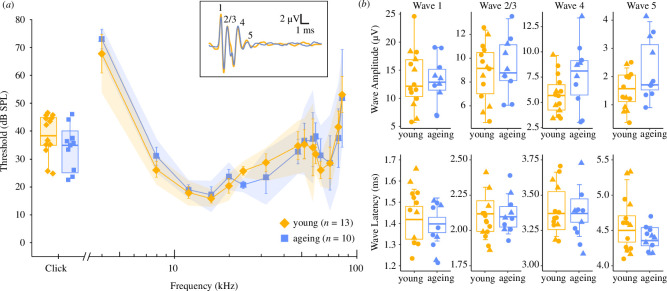
Hearing sensitivity in young and ageing *E. fuscus*. (*a*) Mean ABR-derived thresholds evoked by broadband clicks are shown as boxplots. Curves and error bars represent mean tone-evoked thresholds ± standard error; 95% confidence intervals are indicated as shaded ribbons for each audiogram. Grand mean ABR waveforms (inset) show similar morphology across age groups. (*b*) Amplitudes (top) and latencies (bottom) for waves 1–5 of the click-evoked ABR are represented as boxplots with individual data points for young and ageing male (triangles) and female (circles) bats.

We measured hearing sensitivity thresholds by recording ABRs in 13 young bats ranging in age from 1.2 to 5.9 years (mean age: 3.5 years; mean weight: 17.2 g) and 10 ageing bats from 6.8 to 12.5 years (mean age: 8.2 years; mean weight: 17.0 g). *E. fuscus* are sensitive to sounds from 4 to 84 kHz, with peaks in sensitivity from 12 to 24 kHz and 60 to 72 kHz (figure 1*a*). ABR-derived audiograms for young and ageing *E. fuscus* were similar across the range of frequencies tested, indicating comparable hearing sensitivity across age groups (figure 1*a*). Auditory detection thresholds were not statistically different across age group (*F*_1,18.55 _= 0.66, *p* = 0.43) or sex (*F*_1,17.57 _= 4.09, *p* = 0.06), nor was there a significant interaction effect of age group with frequency (*F*_15,181.56 _= 0.58, *p* = 0.89) or age group with sex (*F*_1,17.57 _= 1.20, *p* = 0.29).

The ABR in *E. fuscus* is characterized by 4–5 biphasic waves (figure 1*a*), following the typical mammalian pattern. In young *E. fuscus*, ABR wave 1, representing activity in the auditory nerve, was typically evoked within approximately 1.4 ms of stimulation (electronic supplementary material, table S1). Waves 2 and 3, representing early brainstem auditory processing centres, often merged to form a single wave approximately 2.1 ms following stimulus onset. Waves 4 and 5, representing higher-order brainstem and midbrain nuclei, occurred within 3.4 and 4.6 ms of stimulus onset, respectively.

The first wave of the ABR is commonly used as an indicator of cochlear integrity, as reduced amplitude and increased latency of ABR wave 1 can reflect early neural contributors to ARHL and may precede threshold elevation [60,61]. Wave 1 amplitudes of the click-evoked ABR showed no significant effects of age group (*F*_1,20.22 _= 0.18, *p* = 0.68), sex (*F*_1,20.22 _= 0.07, *p* = 0.79), or their interaction (*F*_1,20.22 _= 0.67, *p* = 0.42) (electronic supplementary material, table S2). There was a significant interaction of age group across stimulus level on wave 1 amplitudes (*F*_3,63.25 _= 4.93, *p* = 3.9 × 10^−3^); however, *post hoc* analyses showed no significant pairwise differences (electronic supplementary material, table S3). Waves 2/3 through 5 of the click-evoked ABR tended to be of greater amplitude in ageing bats (figure 1*b*), with group differences ranging from 0.47 µV for wave 2/3 to 1.76 µV for wave 4. The amplitudes of ABR waves 4 and 5 showed a significant interaction effect of age and sex (wave 4: *F*_1,20.38 _= 5.26, *p* = 0.03; wave 5: *F*_1,20.31 _= 4.82, *p* = 0.04), in which ageing male bats had significantly larger wave amplitudes relative to young males (wave 4: *p* = 7.0 × 10^−3^, wave 5: *p* = 0.02).

Click-evoked ABRs had slightly faster wave latencies on average in ageing bats relative to young bats (figure 1*b*), but these group differences were small, averaging approximately 0.02 ms for waves 1–4 and 0.20 ms for wave 5. Despite this, wave 4 showed a significant interaction effect of age group and sex on latency (*F*_1,20.35 _= 8.61, *p* = 8.1 × 10^−3^; electronic supplementary material, table S2), in which ageing male bats showed faster wave latencies relative to younger males (*p* = 0.02). Wave 5 onset also occurred earlier in ageing males compared to young (figure 1*b*), but this trend was not significant, likely owing to variation in wave 5 latency among young males.

We additionally explored age-related changes to tone-evoked ABRs for behaviourally relevant frequencies contained within the fundamental sweep of the *E. fuscus* echolocation call: 20–48 kHz [62]. Although ageing bats had slightly larger wave 1 amplitudes evoked by 20–48 kHz tones, these differences were not significant across age groups (electronic supplementary material, table S4). We observed no significant effects of age group or the interactions of age group with level and with sex on the amplitudes or latencies of ABR waves 1–5 for these frequencies (electronic supplementary material, tables S4–S5).

**Figure 2.**
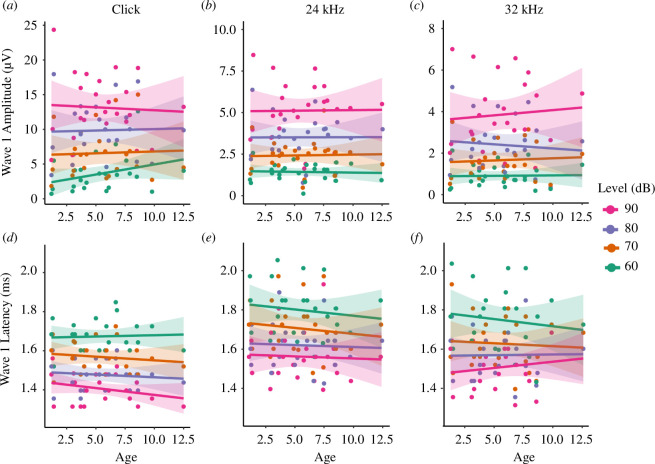
Age effects on *E. fuscus* ABR wave 1 amplitudes (top) and latencies (bottom) evoked by clicks (*a, d*), 24 kHz (*b, e*) and 32 kHz (*c, f*) tones presented from 60 to 90 dB SPL. Lines represent the linear regression of ABR wave amplitudes across the range of tested ages for each stimulus level; shaded ribbons represent 95% confidence intervals for each model.

Because within-group variation of ABRs could potentially overshadow more subtle age-related trends, we assessed the relationship of ABR wave 1 amplitude and latency with chronological age using linear regression models fitted to the data. Wave 1 amplitudes showed a slight negative correlation with advancing age for high-intensity clicks (figure 2*a*; 90 dB click: *r* = −0.05), but not for tones (figure 2*b,c*; 24 kHz at 90 dB: *r* = 0.01, 32 kHz at 90 dB: *r* = 0.08). Wave 1 latencies were positively correlated with age for 32 kHz tones presented at 90 dB (*r* = 0.15), indicating slightly delayed responses among ageing bats relative to young bats. This trend reversed for lower-level (60–70 dB) 32 kHz stimuli, in which latencies reduced slightly with age (figure 2*f*, electronic supplementary material, table S8). However, there was no significant correlation between age and wave 1 amplitudes or latencies evoked by clicks or tones from 20 to 48 kHz (electronic supplementary material, table S8), indicating that age-related changes to the ABR were negligible.

### (b) Outer hair cell functionality is similar among young and ageing bats

**Figure 3.**
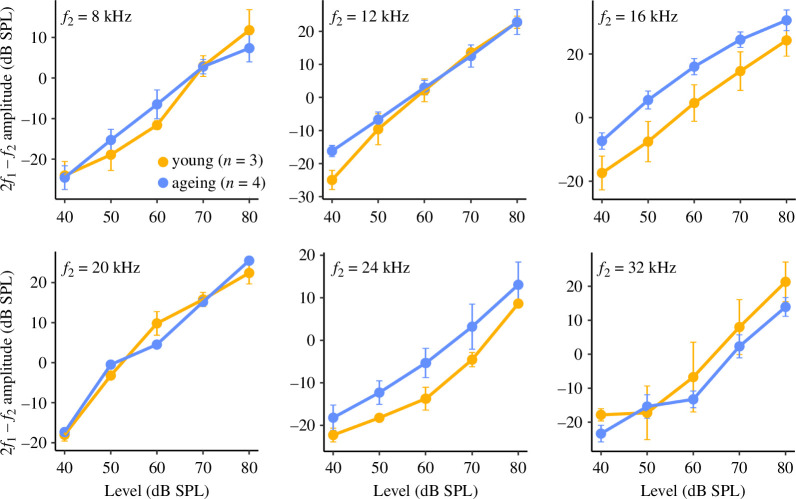
DPOAE amplitudes as a function of stimulus level in young and ageing *E. fuscus*. DPOAE input–output curves represent mean amplitudes of otoacoustic emissions evoked by stimulus frequencies *f_2_* = 8–32 kHz presented from 20 to 80 dB SPL; error bars show standard error of the mean.

To evaluate the functional integrity of the outer hair cells in young and ageing bats, we assessed level-dependent changes to the amplitude of the DPOAE evoked by *f_2_* frequencies from 8 to 32 kHz. In young *E. fuscus*, the DPOAE input–output (I/O) function showed a monotonic increase in amplitude per 10 dB stimulus level increase with a slope approximating 1 (figure 3). Young and ageing bats showed comparable DPOAE I/O functions across the frequencies tested, with no significant effects of age group (*F*_1,180.12_= 1.30, *p* = 0.26) or interactions of age group with frequency (*F*_1,177.01_= 0.46, *p* = 0.50) or with level (*F*_1,177.00_= 0.62, *p* = 0.43) on DPOAE amplitudes. DPOAE I/O functions were shifted upwards in ageing bats for *f_2_* = 16 and 24 kHz (figure 3), indicating level-independent increases to amplitudes of otoacoustic emissions relative to young bats, although these differences were not significant.

### (c) Bat cochlear morphology shows minimal effects of ageing

**Figure 4.**
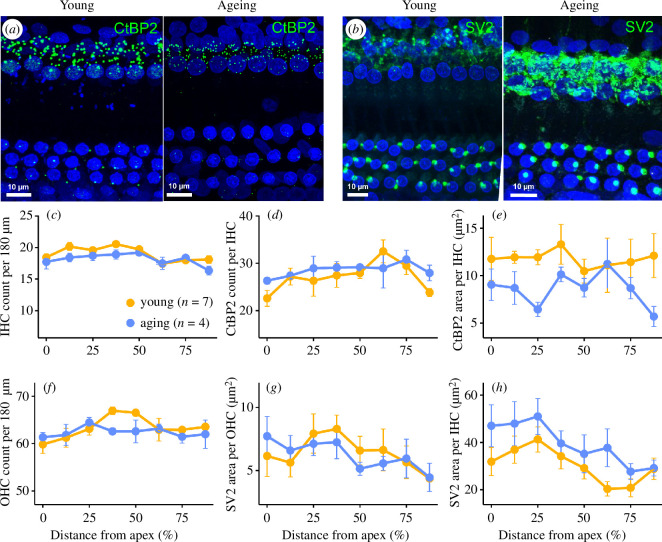
Cochlear immunolabelling in young and ageing *E. fuscus*. Maximum projections of the middle turn of the cochlea from a young (1.4-year-old) and ageing (12.5-year-old) *E. fuscus*. Tissue is labelled with cellular marker DAPI (blue), (*a*) presynaptic ribbon marker CtBP2 (green) and (*b*) efferent terminal marker SV2 (green). (*c*) Inner hair cell counts in young and ageing bats, along with (*d, e*) quantification of presynaptic CtBP2-positive immunopuncta per inner hair cell. (*f*) Outer hair cell counts in young and ageing bats, with quantification of SV2-positive efferent terminals per (*g*) outer and (*h*) inner hair cell. (*c–h*) Data represent mean structural measurements (± standard error) quantified from eight equidistant regions measured as percent total distance from the apex of the cochlea.

Age-related changes to cochlear structural morphology were assessed using whole-mount immunofluorescent preparations from young and ageing *E. fuscus* (figure 4*a,b*). The ageing bat cochlea showed a slight, non-significant reduction in number of inner hair cells (IHCs) compared to young bats (figure 4*c*; *F*_1,127.48_ = 0.61, *p* = 0.44). We observed no age-related variance in the number of presynaptic ribbons (figure 4*d*, *F*_1,53.14_ = 0.05, *p* = 0.83), with young and ageing bats averaging 27–29 ribbons per IHC. However, the overall size of the ribbons, quantified as the total area of CtBP2-positive immunopuncta per IHC, was generally smaller in the ageing bat cochlea (8.6 µm^2^ per IHC) compared to young (12.1 µm^2^ per IHC). Presynaptic ribbons were smallest at locations closer to the apex and base of the ageing bat cochlea (figure 4*e*), but ribbon size was not significantly affected by age group (*F*_1,15.13_=3.99, *p* = 0.06) or the interaction of age group and location along the cochlea (*F*_1,53.08_=0.57, *p* = 0.45).

Additionally, ageing bats showed a slight reduction in the number of outer hair cells (OHCs) at the middle turn of the cochlea approximately 37.5–50.0% distance from the apex compared to young bats (figure 4*f*) but, as with the IHCs, this age difference was not significant (*F*_1,126.67_=2.18, *p* = 0.14). Efferent innervation of the OHCs, quantified as the total area of SV2-positive immunopuncta per cell, was comparable across age groups (figure 4*g*), indicating no significant age-related changes to the medial olivocochlear (OC) system in big brown bats (*F*_1,65.10 _= 0.11, *p* = 0.74). In contrast, the density of efferent innervation of the IHCs, representing the lateral OC terminals, appeared to be enhanced among ageing bats, particularly at the apical low-frequency regions of the cochlea (figure 4*h*). Despite this, the area of SV2-positive efferent terminals per IHC was not significantly different across age groups *(F*_1,17.29 _= 0.32, *p* = 0.58), nor was there an effect of the interaction of age group and location along the cochlea (*F*_1,64.16 _= 0.24, *p* = 0.62).

## 4. Discussion

### (a) Ageing big brown bats retain ‘youthful’ auditory sensitivity

Hearing is one of the most important sensory modalities for echolocating bats because the ability to detect low-intensity returning echoes is essential for biosonar. Further, most echolocating bat species are exceptionally long-lived for their size; therefore, the selective pressures that shape longevity and echolocation in bats may also drive the retention of sufficient hearing capabilities into old age. The typical trajectory of ARHL in most mammals, including humans, is a progressive loss of sensitivity to sound, beginning with high-frequency deficits that extend to low frequencies over time [63]. Here, we investigated the effects of ageing on peripheral auditory sensitivity and cochlear structural integrity in an auditory specialist species, the echolocating big brown bat (*E. fuscus*). We showed that ageing big brown bats retain comparable hearing sensitivity to young bats, demonstrated by similar ABR-derived thresholds in animals ranging from 1.2 to 12.5 years old. The ABR-derived audiogram measured in ageing bats overlapped with the young bat audiogram, extending across the same range of frequencies, with peak sensitivity from 12 to 24 kHz and 60 to 72 kHz. In contrast, mouse models that show ‘normal’ (i.e. not accelerated) ARHL typically exhibit behavioural and physiological sensitivity deficits beginning at 15 months that become severe (threshold elevation of > 60 dB) around 18 months of age (e.g. upon reaching 50% of their maximum 36-month lifespan) [64,65]. We did not observe similar levels of hearing loss in ageing big brown bats, indicating that this long-lived species is resistant to, or exhibits considerably delayed, ARHL.

Additionally, we found that the first wave of the ABR, representing the auditory nerve response and typically considered a metric for cochlear functional integrity, showed minimal non-significant amplitude reductions across age groups. Cochlear ageing in humans is correlated with a progressive decline in hair cells, spiral ganglion cells and auditory nerve fibres [8,66,67], a phenomenon that is mirrored in animal models for ARHL [60,61,63,64]. These structural changes are reflected physiologically as reductions to suprathreshold amplitudes of ABR wave 1, which may precede evidence for threshold elevation [60]. In rodents, age-related reduction to ABR wave amplitudes can be profound, with aged animals commonly showing more than 65% reduction in wave 1 of the click-evoked response by 18 months [60,64,68]. In contrast, suprathreshold wave 1 amplitudes appeared robust to ageing in big brown bats, with comparatively small (approximately 11%) decrements of the click-evoked response in the oldest bats. Further, there were no significant age-related differences in wave 1 amplitudes evoked by frequencies characteristic of the species-specific echolocation call, indicating that ageing big brown bats may be resistant to auditory functional declines typically associated with natural senescence.

The later waves of the ABR showed evidence for sex-biased amplitude enhancements with age, in which wave 4 amplitudes increased by approximately 160% and wave 5 more than doubled in ageing male bats compared to young males. This enhancement was accompanied by a non-significant decrease in ABR wave 1 of approximately 0.5 µV (compared to the 0.16 µV decrement in ageing female bats that did not show late wave enhancements of such magnitude). In ageing rodents and humans, reductions to ABR wave 1 are correlated with stable or increased amplitudes of the later waves, which suggests central gain as a compensatory response to age-related deterioration of cochlear input [69–72]. Although the reduction to wave 1 in *E. fuscus* was not statistically significant, even a small modification to peripheral hearing capability may be biologically significant to echolocating species that require acute sensitivity to detect low-intensity echoes. The observed changes to ABR waves in ageing male bats could represent similar compensatory activity in brainstem and midbrain auditory processing centres to maintain sensitivity despite reductions in the cochlear response.

Healthy cochlear function is characterized by compressive, nonlinear amplification driven by the active electro-motile properties of the OHCs, which confers greater sensitivity and sharper frequency tuning [73,74]. OHC loss often precedes IHC loss in the ageing mammalian cochlea, and it can reduce auditory sensitivity independent of the effects of IHC loss [8,64,75]. Age-related changes to OHC function in humans and rodent models for hearing loss are correlated with reduced DPOAEs and shallow, right-shifted growth functions indicating reduced efficacy of the cochlear amplifier [76,77]. In contrast, OHC function in *E. fuscus* was unaffected by age, with no significant age-related changes to DPOAE amplitudes across frequency or level. Comparable DPOAE growth functions for frequencies from 8 to 32 kHz in young and ageing bats further indicate that *E. fuscus* maintains robust cochlear response properties into old age, which may support continued sensitivity to echolocation signals.

### (b) Bat cochlear structures show signs of noise exposure, but not ageing

The peripheral auditory system in mammals is especially susceptible to the cumulative effects of natural senescence and noise damage over a lifetime of exposure to sound. ARHL is commonly associated with the loss of cochlear hair cells, with OHCs being particularly susceptible [8,67]; however, mounting evidence suggests that the perceptual deficits associated with ARHL (e.g. threshold elevation, difficulty listening in noise) are associated with degeneration of the synaptic interface between IHCs and afferent fibres [10,11,60,61]. Ageing CBA/CaJ mice—a strain used to model a normal, human-like trajectory of ARHL, show up to 50% loss of IHCs from the high-frequency basal region of the cochlea and up to 70% loss of OHCs towards the low-frequency apical region [64]. Additionally, ageing mice lose nearly half of their cochlear ribbon synapses prior to showing evidence for hair cell loss and deafferentation [10,60,78].

In the present study, we found no significant evidence for age-related loss of IHCs or OHCs in big brown bats. Further, we observed no significant changes to the number of presynaptic ribbons mediating the afferent auditory pathway in ageing bats. However, ribbons were slightly reduced in size among ageing bats, potentially indicating senescent changes to cochlear structures. In contrast, synaptopathy in the ageing mouse cochlea is associated with enlargement of the remaining IHC ribbons, perhaps as a compensatory mechanism for reduced sensory inputs but also potentially contributing to maladaptive hyperacusis-like responses [79,80]. Small ribbon sizes in ageing big brown bats may reflect the consequences of a history of exposure to loud sounds, which has been correlated with ribbon size reduction in guinea pigs [81].

Although there were no significant age-related differences in efferent innervation of the OHCs by medial OC neurons, we observed a trend for slightly increased efferent innervation of the IHCs by lateral OC neurons in ageing bats. The OC system enhances auditory detection of signals in noise and may confer protection against acoustic injury via top–down control of the cochlear response (reviewed in [82,83]). The lateral OC system is composed of unmyelinated neurons that originate in and around the lateral superior olive and terminate on the type I afferent fibres that synapse with IHCs [82]. Compared to the medial OC, little is known about the mechanism of action of the lateral OC [84]. Although the lateral OC is hypothesized to prevent acoustic overexposure by protecting against excitotoxicity at the IHC–afferent synapse [85–87], noise- and experience-dependent plasticity of lateral OC neurons are only beginning to be understood [88,89]. A greater density of lateral OC efferent terminals in ageing bats could be another structural indicator of a history of noise exposure that is suggestive of enhanced efferent modulation to prevent noise-induced cochlear damage.

### (c) Echolocating bats as an informative model for resistance to ageing and noise

Echolocating bats represent a powerful research model for investigating mechanisms that may protect against noise- and age-related auditory damage: naturally behaving bats regularly emit intense biosonar signals that subject the auditory system to potentially damaging self-generated sound while simultaneously requiring acute sensitivity to low-intensity returning echoes. Hearing loss could prove fatal to an echolocating bat in the wild, as the inability to detect quiet echoes would prevent effective foraging, navigation and obstacle avoidance. Although echolocation and intense sound exposure are inextricably linked in bats, the combined physiological and histological results of this study suggest that there is no functional loss of hearing in ageing big brown bats.

A limitation of this study is that we were unable to sample bats at the higher end of the maximum longevity for this species (e.g. bats nearing 19 years of age). Therefore, it is possible that we were not able to fully characterize auditory changes that may occur in this species at very old ages. Despite this, we demonstrate that ageing big brown bats retain ‘youthful’ physiological hearing sensitivity for up to 12.5 years of life (approximately 66% of the species’ maximum lifespan [35]). Surveys of wild *E. fuscus* populations reveal that approximately 20% of bats survive past 6 years of age and even fewer (3–6%) survive beyond 12 years [35,36,40], indicating that comparable auditory sensitivity in bats from 1.2 to 12.5 years old represents a reasonable assessment of hearing to the end of the natural lifespan in this species. In contrast, a recent study using Egyptian fruit bats revealed that not all bat species are resistant to ARHL, with ageing fruit bats showing high- to low-frequency hearing deficits comparable to those observed in ageing humans [30]. Egyptian fruit bats averaging 12 years of age (approximately 50% of the species’ maximum lifespan [34]) showed 10 dB threshold elevations relative to younger bats [30], indicating greater susceptibility to ARHL compared to big brown bats.

Cross-species variability in susceptibility to ARHL may reflect different selective pressures experienced by bats depending on what sensory channels are available to guide behaviours critical for survival. For example, Egyptian fruit bats are able to integrate lingual echolocation with vision during foraging and navigation, with recent evidence suggesting that this species preferentially uses visual over acoustic cues [32]. In contrast, frequency-modulated echoes from self-generated biosonar signals provide the primary sensory cue that enables big brown bats to pursue prey [90]. As such, it is possible that aerial-hawking species like *E. fuscus* possess auditory specializations to protect against hearing loss (reviewed in [91]). Variable susceptibility to noise exposure in echolocating versus non-echolocating (i.e. visually dominant) bat species presents a complementary view [31] and highlights the value of comparing diverse species to gain a greater understanding of what mechanisms may support cochlear integrity in some, but not all, species.

In bats, the mechanisms that support exceptional longevity may also play a role in protecting against cochlear damage. ARHL is linked to peripheral sensorineural damage, including loss of cochlear hair cells and loss of afferent and efferent neurons, as well as degeneration of the stria vascularis, an energetically active structure that maintains the endocochlear potential. In particular, the accumulation of reactive oxygen species (ROS) and subsequent ROS-driven mitochondrial dysfunction have been implicated in cochlear ageing [92,93]. Many bat species appear to be resistant to oxidative stress [94,95], an attribute that supports homeostatic processes over a long lifespan and may similarly protect against age-related accumulation of oxidative damage in the cochlea. Despite this, ARHL in the Egyptian fruit bat is correlated with clear structural markers of strial atrophy [30]. Careful investigation of the stria vascularis in ageing big brown bats is a key next step to evaluate whether this species shows resistance to age-related strial degeneration.

The protective factors that may underlie resistance to noise in bats remain unknown; however, the auditory efferent system presents an intriguing avenue for future research. Previous morphological studies have revealed that the efferent OC system is hypertrophic in bats, with some species showing nearly three times the number of efferent OC neurons observed in mice [96–98]. Here, we observed similar OC enhancement in *E. fuscus,* with young bats showing twice the area of efferent innervation per inner and outer hair cell compared to young mice [64]. Strong top-down efferent modulation of the cochlear response to sound has been observed in bats living in noisy roosts [99] and during call production [100] as a protective mechanism against self-generated and background sounds. Further physiological and behavioural investigation of efferent function in young and ageing bats, particularly those that utilize active and passive listening in noisy environments, will extend our understanding of the mechanisms that allow these long-lived auditory specialists to resist the damaging effects of intense sounds.

## Data Availability

Methylation data used for age estimation are available on the NCBI Gene Expression Omnibus repository, accession number GSE274777. Data and code to replicate the analyses of this study are available on Figshare [101]. Supplementary material is available online [102].

## References

[1] Foeller E , Kössl M . 2000 Mechanical adaptations for echolocation in the cochlea of the bat Hipposideros lankadiva. J. Comp. Physiol. A: Sensory, Neural, Behav. Physiol. **186** , 859–870. (10.1007/s003590000139) 11085639

[2] Russell IJ , Kössl M . 1999 Micromechanical responses to tones in the auditory fovea of the greater mustached bat’s cochlea. J. Neurophysiol. **82** , 676–686. (10.1152/jn.1999.82.2.676) 10444665

[3] Covey E . 2005 Neurobiological specializations in echolocating bats. Anat. Rec. **287A** , 1103–1116. (10.1002/ar.a.20254) 16217809

[4] Holderied MW , Korine C , Fenton MB , Parsons S , Robson S , Jones G . 2005 Echolocation call intensity in the aerial hawking bat Eptesicus bottae (Vespertilionidae) studied using stereo videogrammetry. J. Exp. Biol. **208** , 1321–1327. (10.1242/jeb.01528) 15781892

[5] Brinkløv S , Jakobsen L , Ratcliffe JM , Kalko EKV , Surlykke A . 2011 Echolocation call intensity and directionality in flying short-tailed fruit bats, Carollia perspicillata (Phyllostomidae). J. Acoust. Soc. Am. **129** , 427–435. (10.1121/1.3519396) 21303022

[6] Jones G , Holderied MW . 2007 Bat echolocation calls: adaptation and convergent evolution. Proc. R. Soc. B **274** , 905–912. (10.1098/rspb.2006.0200) PMC1919403 17251105

[7] Surlykke A , Kalko EKV . 2008 Echolocating bats cry out loud to detect their prey. PLoS One **3** , e2036. (10.1371/journal.pone.0002036) 18446226 PMC2323577

[8] Wu PZ , O’Malley JT , Gruttola V , Charles Liberman M . 2020 Age-related hearing loss is dominated by damage to inner ear sensory cells, not the cellular battery that powers them. J. Neurosci. **40** , 6357–6366. (10.1523/JNEUROSCI.093720.2020) 32690619 PMC7424870

[9] Wong ACY , Ryan AF . 2015 Mechanisms of sensorineural cell damage, death and survival in the cochlea. Front. Aging Neurosci. **7** , 58. (10.3389/fnagi.2015.00058) 25954196 PMC4404918

[10] Kujawa SG , Liberman MC . 2015 Synaptopathy in the noise-exposed and aging cochlea: primary neural degeneration in acquired sensorineural hearing loss. Hear. Res. **330** , 191–199. (10.1016/j.heares.2015.02.009) 25769437 PMC4567542

[11] Parthasarathy A , Kujawa SG . 2018 Synaptopathy in the aging cochlea: characterizing early-neural deficits in auditory temporal envelope processing. J. Neurosci. **38** , 7108–7119. (10.1523/JNEUROSCI.3240-17.2018) 29976623 PMC6596096

[12] Bao J , Ohlemiller KK . 2010 Age-related loss of spiral ganglion neurons. Hear. Res. **264** , 93–97. (10.1016/j.heares.2009.10.009) 19854255 PMC2868093

[13] Lang H , Noble KV , Barth JL , Rumschlag JA , Jenkins TR , Storm SL , Eckert MA , Dubno JR , Schulte BA . 2023 The stria vascularis in mice and humans is an early site of age-related cochlear degeneration, macrophage dysfunction, and inflammation. J. Neurosci. **43** , 5057–5075. (10.1523/JNEUROSCI.2234-22.2023) 37268417 PMC10324995

[14] Seidman MD , Ahmad N , Joshi D , Seidman J , Thawani S , Quirk WS . 2004 Age-related hearing loss and its association with reactive oxygen species and mitochondrial DNA damage. Acta Otolaryngol. **124** , 16–24. (10.1080/03655230410017823) 15219042

[15] Cheng AG , Cunningham LL , Rubel EW . 2005 Mechanisms of hair cell death and protection. Curr. Opin. Otolaryngol. Head Neck Surg. **13** , 343–348. (10.1097/01.moo.0000186799.45377.63) 16282762

[16] Yamasoba T , Lin FR , Someya S , Kashio A , Sakamoto T , Kondo K . 2013 Current concepts in age-related hearing loss: epidemiology and mechanistic pathways. Hear. Res. **303** , 30–38. (10.1016/j.heares.2013.01.021) 23422312 PMC3723756

[17] Brunet-Rossinni AK , Austad SN . 2004 Ageing studies on bats: a review. Biogerontology **5** , 211–222. (10.1023/B:BGEN.0000038022.65024.d8) 15314271

[18] Dammann P . 2017 Slow aging in mammals—lessons from African mole-rats and bats. Semin. Cell Dev. Biol. **70** , 154–163. (10.1016/j.semcdb.2017.07.006) 28698112

[19] Munshi-South J , Wilkinson GS . 2010 Bats and birds: exceptional longevity despite high metabolic rates. Ageing Res. Rev. **9** , 12–19. (10.1016/j.arr.2009.07.006) 19643206

[20] Wilkinson GS , Adams DM . 2019 Recurrent evolution of extreme longevity in bats. Biol. Lett. **15** , 20180860. (10.1098/rsbl.2018.0860) 30966896 PMC6501359

[21] Cooper LN *et al* . 2024 Bats as instructive animal models for studying longevity and aging. Ann. N. Y. Acad. Sci. (10.1111/nyas.15233) PMC11580778 39365995

[22] Baudry M , DuBrin R , Beasley L , Leon M , Lynch G . 1986 Low levels of calpain activity in Chiroptera brain: implications for mechanisms of aging. Neurobiol. Aging **7** , 255–258. (10.1016/0197-4580(86)90004-7) 3018604

[23] Conde-Pérezprina JC , Luna-López A , González-Puertos VY , Zenteno-Savín T , León-Galván MA , Königsberg M . 2012 DNA MMR systems, microsatellite instability and antioxidant activity variations in two species of wild bats: Myotis velifer and Desmodus rotundus, as possible factors associated with longevity. Age (Omaha) **34** , 1473–1492. (10.1007/s11357-012-9399-5) PMC3528369 22453932

[24] Kacprzyk J , Hughes GM , Palsson-McDermott EM , Quinn SR , Puechmaille SJ , O’Neill LAJ , Teeling EC . 2017 A potent anti-inflammatory response in bat macrophages may be linked to extended longevity and viral tolerance. Acta Chiropterol. **19** , 219–228. (10.3161/15081109ACC2017.19.2.001)

[25] Banerjee A , Baker ML , Kulcsar K , Misra V , Plowright R , Mossman K . 2020 Novel insights into immune systems of bats. Front. Immunol. **11** , 26. (10.3389/fimmu.2020.00026) 32117225 PMC7025585

[26] Ball HC , Levari-Shariati S , Cooper LN , Aliani M . 2018 Comparative metabolomics of aging in a long-lived bat: insights into the physiology of extreme longevity. PLoS One **13** , e0196154. (10.1371/journal.pone.0196154) 29715267 PMC5929510

[27] Ungvari Z , Buffenstein R , Austad SN , Podlutsky A , Kaley G , Csiszar A . 2008 Oxidative stress in vascular senescence: lessons from successfully aging species. Front. Biosci. **13** , 5056–5070. (10.2741/3064) 18508570

[28] Huang Z , Whelan CV , Foley NM , Jebb D , Touzalin F , Petit EJ , Puechmaille SJ , Teeling EC . 2019 Longitudinal comparative transcriptomics reveals unique mechanisms underlying extended healthspan in bats. Nat. Ecol. Evol. **3** , 1110–1120. (10.1038/s41559-019-0913-3) 31182815

[29] Weinberg MM , Retta NA , Schrode KM , Screven LA , Peterson JL , Moss CF , Sterbing S , Lauer AM . 2021 Deafness in an auditory specialist, the big brown bat (Eptesicus fuscus). Hear. Res. **412** , 108377. (10.1016/j.heares.2021.108377) 34735823

[30] Tarnovsky YC , Taiber S , Nissan Y , Boonman A , Assaf Y , Wilkinson GS , Avraham KB , Yovel Y . 2023 Bats experience age-related hearing loss (presbycusis). Life Sci. Alliance **6** , e202201847. (10.26508/lsa.202201847) 36997281 PMC10067528

[31] Liu Z *et al* . 2021 Cochlear hair cells of echolocating bats are immune to intense noise. J. Genet. Genomics **48** , 984–993. (10.1016/j.jgg.2021.06.007) 34393089

[32] Danilovich S , Yovel Y . 2019 Integrating vision and echolocation for navigation and perception in bats. Sci. Adv. **5** , eaaw6503. (10.1126/sciadv.aaw6503) 31249874 PMC6594759

[33] Hulgard K , Moss CF , Jakobsen L , Surlykke A . 2016 Big brown bats (Eptesicus fuscus) emit intense search calls and fly in stereotyped flight paths as they forage in the wild. J. Exp. Biol. **219** , 334–340. (10.1242/jeb.128983) 26596537

[34] Wilkinson GS , South JM . 2002 Life history, ecology and longevity in bats. Aging Cell **1** , 124–131. (10.1046/j.1474-9728.2002.00020.x) 12882342

[35] Hitchcock HB . 1965 Twenty-three years of bat banding in Ontario and Quebec. Can. Field. Nat. **79** , 4–14. (10.5962/p.342362)

[36] O’Shea TJ , Ellison LE , Stanley TR . 2011 Adult survival and population growth rate in Colorado big brown bats (Eptesicus fuscus). J. Mammal. **92** , 433–443. (10.1644/10-MAMM-A-162.1)

[37] Simmons AM , Hom KN , Warnecke M , Simmons JA . 2016 Broadband noise exposure does not affect hearing sensitivity in big brown bats (Eptesicus fuscus) . J. Exp. Biol. **219** , 1031–1040. (10.1242/jeb.135319) 27030779

[38] Simmons AM , Hom KN , Simmons JA . 2017 Big brown bats (Eptesicus fuscus) maintain hearing sensitivity after exposure to intense band-limited noise . J. Acoust. Soc. Am. **141** , 1481–1489. (10.1121/1.4976820) 28372082

[39] Hom KN , Linnenschmidt M , Simmons JA , Simmons AM . 2016 Echolocation behavior in big brown bats is not impaired after intense broadband noise exposures. J. Exp. Biol. (10.1242/jeb.143578) 27510961

[40] Goehring HH . 1972 Twenty-year study of Eptesicus fuscus in Minnesota. J. Mammal. **53** , 201. (10.2307/1378850)

[41] Mills RS , Barrett GW , Farrell MP . 1975 Population dynamics of the big brown bat (Eptesicus fuscus) in Southwestern Ohio. J. Mammal. **56** , 591–604. (10.2307/1379471)

[42] Kurta A , Baker RH . 1990 Eptesicus fuscus. Mamm. Species. **356** , 1. (10.2307/3504258)

[43] Flurkey K , Currer JM , Harrison DE . 2007 Mouse models in aging research. In The mouse in biomedical research: history (eds JG Fox , MT Davisson , FW Quimby , SW Barthold , CE Newcomer , AL Smith ), pp. 637–672. Burlington, MA: Elsevier. (10.1016/B978-012369454-6/50074-1)

[44] Burke K , Screven LA , Kobrina A , Charlton PE , Schrode K , Villavisanis DF , Dent ML , Lauer AM . 2022 Effects of noise exposure and aging on behavioral tone detection in quiet and noise by mice. eNeuro **9** , 1–15. (10.1523/ENEURO.0391-21.2022) PMC9191765 35613853

[45] Guimaraes P , Zhu X , Cannon T , Kim S , Frisina RD . 2004 Sex differences in distortion product otoacoustic emissions as a function of age in CBA mice. Hear. Res. **192** , 83–89. (10.1016/j.heares.2004.01.013) 15157966

[46] Kim MJ *et al* . 2023 Sex differences in body composition, voluntary wheel running activity, balance performance, and auditory function in CBA/CaJ mice across the lifespan. Hear. Res. **428** , 108684. (10.1016/j.heares.2022.108684) 36599258 PMC11446250

[47] Ohlemiller KK , Wright JS , Heidbreder AF . 2000 Vulnerability to noise-induced hearing loss in ‘middle-aged and young adult mice: a dose–response approach in CBA, C57BL, and BALB inbred strains. Hear. Res. **149** , 239–247. (10.1016/s0378-5955(00)00191-x) 11033262

[48] Park CR , Willott JF , Walton JP . 2024 Age-related changes of auditory sensitivity across the life span of CBA/CaJ mice. Hear. Res. **441** , 108921. (10.1016/j.heares.2023.108921) 38042127 PMC10843596

[49] Capshaw G , Vicencio-Jimenez S , Screven LA , Burke K , Weinberg MM , Lauer AM . 2022 Physiological evidence for delayed age-related hearing loss in two long-lived rodent species (Peromyscus leucopus and P. californicus). J. Assoc. Res. Otolaryngol. **23** , 617–631. (10.1007/s10162-022-00860-4) 35882705 PMC9613845

[50] Guo Z , Wang M , Tian G , Burger J , Gochfeld M , Yang CS . 1993 Age- and gender-related variations in the activities of drug-metabolizing and antioxidant enzymes in the white-footed mouse (Peromyscus leucopus). Growth. Dev. Aging **57** , 85–100. 8495997

[51] Lin Z , Kajigaya S , Feng X , Chen J , Young NS . 2017 Hematopoietic aging biomarkers in Peromyscus leucopus mice. J. Aging Sci. **5** , 169. (10.4172/2329-8847.1000169) 28620625 PMC5469418

[52] Wilkinson GS *et al* . 2021 DNA methylation predicts age and provides insight into exceptional longevity of bats. Nat. Commun. **12** , 1615. (10.1038/s41467-021-21900-2) 33712580 PMC7955057

[53] Arneson A *et al* . 2022 A mammalian methylation array for profiling methylation levels at conserved sequences. Nat. Commun. **13** , 783. (10.1038/s41467-022-28355-z) 35145108 PMC8831611

[54] Brittan-Powell EF , Dooling RJ , Gleich O . 2002 Auditory brainstem responses in adult budgerigars (Melopsittacus undulatus). J. Acoust. Soc. Am. **112** , 999–1008. (10.1121/1.1494807) 12243189

[55] Brittan-Powell EF , Christensen-Dalsgaard J , Tang YZ , Carr CE , Dooling RJ . 2010 The auditory brainstem response in two lizard species. J. Acoust. Soc. Am. **128** , 787–794. (10.1121/1.3458813) 20707448 PMC2933256

[56] Kim YH , Schrode KM , Lauer AM . 2022 Auditory brainstem response (ABR) measurements in small mammals. In Developmental, physiological, and functional neurobiology of the inner ear (ed. AK Groves ), pp. 357–375, vol. 176. New York, NY: Humana. (10.1007/978-1-0716-2022-9_16)

[57] Burke K , Burke M , Lauer AM . 2023 Auditory brainstem response (ABR) waveform analysis program. MethodsX **11** , 102414. (10.1016/j.mex.2023.102414) 37846351 PMC10577057

[58] R Core Team . 2020 R: a language and environment for statistical computing. Vienna, Austria: R Foundation for Statistical Computing. See https://www.r-project.org/.

[59] Schindelin J *et al* . 2012 Fiji: an open-source platform for biological-image analysis. Nat. Methods **9** , 676–682. (10.1038/nmeth.2019) 22743772 PMC3855844

[60] Sergeyenko Y , Lall K , Liberman MC , Kujawa SG . 2013 Age-related cochlear synaptopathy: an early-onset contributor to auditory functional decline. J. Neurosci. **33** , 13686–13694. (10.1523/JNEUROSCI.1783-13.2013) 23966690 PMC3755715

[61] Kujawa SG , Liberman MC . 2009 Adding insult to injury: cochlear nerve degeneration after ‘temporary’ noise-induced hearing loss. J. Neurosci. **29** , 14077–14085. (10.1523/JNEUROSCI.2845-09.2009) 19906956 PMC2812055

[62] Simmons JA , Fenton MB , O’Farrell MJ . 1979 Echolocation and pursuit of prey by bats. Science **203** , 16–21. (10.1126/science.758674) 758674

[63] Ohlemiller KK , Frisina RD . 2008 Age-related hearing loss and its cellular and molecular bases. In Auditory trauma, protection (eds J Schacht , AN Popper , RR Fay ), pp. 145–194. Boston, MA: Springer. (10.1007/978-0-387-72561-1_6)

[64] Kobrina A *et al* . 2020 Linking anatomical and physiological markers of auditory system degeneration with behavioral hearing assessments in a mouse (Mus musculus) model of age-related hearing loss. Neurobiol. Aging **96** , 87–103. (10.1016/j.neurobiolaging.2020.08.012) 32950782 PMC8080312

[65] Zheng QY , Johnson KR , Erway LC . 1999 Assessment of hearing in 80 inbred strains of mice by ABR threshold analyses. Hear. Res. **130** , 94–107. (10.1016/s0378-5955(99)00003-9) 10320101 PMC2855304

[66] Makary CA , Shin J , Kujawa SG , Liberman MC , Merchant SN . 2011 Age-related primary cochlear neuronal degeneration in human temporal bones. J. Assoc. Res. Otolaryngol. **12** , 711–717. (10.1007/s10162-011-0283-2) 21748533 PMC3214241

[67] Wu PZ , Liberman LD , Bennett K , de Gruttola V , O’Malley JT , Liberman MC . 2019 Primary neural degeneration in the human cochlea: evidence for hidden hearing loss in the aging ear. Neuroscience **407** , 8–20. (10.1016/j.neuroscience.2018.07.053) 30099118 PMC6369025

[68] Alvarado JC , Fuentes-Santamaría V , Gabaldón-Ull MC , Blanco JL , Juiz JM . 2014 Wistar rats: a forgotten model of age-related hearing loss. Front. Aging Neurosci. **6** , 29. (10.3389/fnagi.2014.00029) 24634657 PMC3942650

[69] Schrode KM , Muniak MA , Kim YH , Lauer AM . 2018 Central compensation in auditory brainstem after damaging noise exposure. eNeuro **5** , 1–19. (10.1523/ENEURO.0250-18.2018) PMC6096756 30123822

[70] Johannesen PT , Lopez-Poveda EA . 2021 Age-related central gain compensation for reduced auditory nerve output for people with normal audiograms, with and without tinnitus. i. Sci. **24** , 102658. (10.1016/j.isci.2021.102658) PMC8192693 34151241

[71] Cai R , Montgomery SC , Graves KA , Caspary DM , Cox BC . 2018 The FBN rat model of aging: investigation of ABR waveforms and ribbon synapse changes. Neurobiol. Aging **62** , 53–63. (10.1016/j.neurobiolaging.2017.09.034) 29107847 PMC5743589

[72] Rumschlag JA , McClaskey CM , Dias JW , Kerouac LB , Noble KV , Panganiban C , Lang H , Harris KC . 2022 Age-related central gain with degraded neural synchrony in the auditory brainstem of mice and humans. Neurobiol. Aging **115** , 50–59. (10.1016/j.neurobiolaging.2022.03.014) 35468552 PMC9153923

[73] Dallos P . 2008 Cochlear amplification, outer hair cells and prestin. Curr. Opin. Neurobiol. **18** , 370–376. (10.1016/j.conb.2008.08.016) 18809494 PMC2630119

[74] Fettiplace R . 2020 Diverse mechanisms of sound frequency discrimination in the vertebrate cochlea. Trends Neurosci. **43** , 88–102. (10.1016/j.tins.2019.12.003) 31954526 PMC7015066

[75] Frisina RD , Zhu X . 2010 Auditory sensitivity and the outer hair cell system in the CBA mouse model of age-related hearing loss. Open Access Anim. Physiol. **2** , 9–16. (10.2147/OAAP.S7202) 21866215 PMC3159169

[76] Parham K . 1997 Distortion product otoacoustic emissions in the C57BL/6J mouse model of age-related hearing loss. Hear. Res. **112** , 216–234. (10.1016/s0378-5955(97)00124-x) 9367243

[77] Ueberfuhr MA , Fehlberg H , Goodman SS , Withnell RH . 2016 A DPOAE assessment of outer hair cell integrity in ears with age-related hearing loss. Hear. Res. **332** , 137–150. (10.1016/j.heares.2015.11.006) 26631688

[78] Xiong W , Yu S , Liu K , Gong S . 2020 Loss of cochlear ribbon synapses in the early stage of aging causes initial hearing impairment. Am. J. Transl. Res. **12** , 7354–7366. 33312372 PMC7724364

[79] Stamataki S , Francis HW , Lehar M , May BJ , Ryugo DK . 2006 Synaptic alterations at inner hair cells precede spiral ganglion cell loss in aging C57BL/6J mice. Hear. Res. **221** , 104–118. (10.1016/j.heares.2006.07.014) 17005343

[80] Jeng JY , Ceriani F , Olt J , Brown SDM , Holley MC , Bowl MR , Johnson SL , Marcotti W . 2020 Pathophysiological changes in inner hair cell ribbon synapses in the ageing mammalian cochlea. J. Physiol. **598** , 4339–4355. (10.1113/JP280018) 32710572 PMC7612121

[81] Shi L , Liu L , He T , Guo X , Yu Z , Yin S , Wang J . 2013 Ribbon synapse plasticity in the cochleae of guinea pigs after noise-induced silent damage. PLoS One **8** , e81566. (10.1371/journal.pone.0081566) 24349090 PMC3857186

[82] Guinan JJ . 1996 Physiology of olivocochlear efferents. In The cochlea, vol. 8 (eds P Dallos , A Popper , R Fay ), pp. 435–502. New York, NY: Springer-Verlag.

[83] Guinan JJ . 2018 Olivocochlear efferents: their action, effects, measurement and uses, and the impact of the new conception of cochlear mechanical responses. Hear. Res. **362** , 38–47. (10.1016/j.heares.2017.12.012) 29291948 PMC5911200

[84] Fuchs PA , Lauer AM . 2019 Efferent Inhibition of the cochlea. Cold Spring Harb. Perspect. Med. **9** , 1–16. (10.1101/cshperspect.a033530) PMC6496333 30082454

[85] Ruel J , Wang J , Rebillard G , Eybalin M , Lloyd R , Pujol R , Puel JL . 2007 Physiology, pharmacology and plasticity at the inner hair cell synaptic complex. Hear. Res. **227** , 19–27. (10.1016/j.heares.2006.08.017) 17079104

[86] Darrow KN , Maison SF , Liberman MC . 2007 Selective removal of lateral olivocochlear efferents increases vulnerability to acute acoustic injury. J. Neurophysiol. **97** , 1775–1785. (10.1152/jn.00955.2006) 17093118 PMC1805782

[87] Groff JA , Liberman MC . 2003 Modulation of cochlear afferent response by the lateral olivocochlear system: activation via electrical stimulation of the inferior colliculus. J. Neurophysiol. **90** , 3178–3200. (10.1152/jn.00537.2003) 14615429

[88] Wu JS , Yi E , Manca M , Javaid H , Lauer AM , Glowatzki E . 2020 Sound exposure dynamically induces dopamine synthesis in cholinergic LOC efferents for feedback to auditory nerve fibers. eLife **9** , 1–27. (10.7554/eLife.52419) PMC7043886 31975688

[89] Frank MM , Sitko AA , Suthakar K , Torres Cadenas L , Hunt M , Yuk MC , Weisz CJC , Goodrich LV . 2023 Experience-dependent flexibility in a molecularly diverse central-to-peripheral auditory feedback system. eLife **12** , e83855. (10.7554/eLife.83855) 36876911 PMC10147377

[90] Moss CF , Surlykke A . 2010 Probing the natural scene by echolocation in bats. Front. Behav. Neurosci. **4** , 1–16. (10.3389/fnbeh.2010.00033) 20740076 PMC2927269

[91] Simmons AM , Simmons JA . 2024 Echolocating bats have evolved decreased susceptibility to noise-induced temporary hearing losses. J. Assoc. Res. Otolaryngol. **25** , 229–238. (10.1007/s10162-024-00941-6) 38565735 PMC11150213

[92] Staecker H , Zheng QY , Van De Water TR . 2001 Oxidative stress in aging in the C57B16/J mouse cochlea. Acta Otolaryngol. **121** , 666–672. (10.1080/00016480152583593) 11678164 PMC2862210

[93] Jiang H , Talaska AE , Schacht J , Sha SH . 2007 Oxidative imbalance in the aging inner ear. Neurobiol. Aging **28** , 1605–1612. (10.1016/j.neurobiolaging.2006.06.025) 16920227 PMC2453750

[94] Salmon AB , Leonard S , Masamsetti V , Pierce A , Podlutsky AJ , Podlutskaya N , Richardson A , Austad SN , Chaudhuri AR . 2009 The long lifespan of two bat species is correlated with resistance to protein oxidation and enhanced protein homeostasis. FASEB J. **23** , 2317–2326. (10.1096/fj.08-122523) 19244163 PMC2704589

[95] Brunet-Rossinni AK . 2004 Reduced free-radical production and extreme longevity in the little brown bat (Myotis lucifugus) versus two non-flying mammals. Mech. Ageing Dev. **125** , 11–20. (10.1016/j.mad.2003.09.003) 14706233

[96] Bishop AL , Henson OW . 1987 The efferent cochlear projections of the superior olivary complex in the mustached bat. Hear. Res. **31** , 175–182. (10.1016/0378-5955(87)90124-9) 3446674

[97] Xie DH , Henson MM , Bishop AL , Henson OW . 1993 Efferent terminals in the cochlea of the mustached bat: quantitative data. Hear. Res. **66** , 81–90. (10.1016/0378-5955(93)90262-y) 7682545

[98] Vater M , Lenoir M , Pujol R . 1992 Ultrastructure of the horseshoe bat’s organ of corti. II.Transmission electron microscopy. J. Comp. Neurol. **318** , 380–391. (10.1002/cne.903180404) 1578009

[99] Xie DH , Henson OW . 1998 Tonic efferent-induced cochlear damping in roosting and echolocating mustached bats. Hear. Res. **124** , 60–68. (10.1016/s0378-5955(98)00122-1) 9822902

[100] Goldberg RL , Henson OW . 1998 Changes in cochlear mechanics during vocalization: evidence for a phasic medial efferent effect. Hear. Res. **122** , 71–81. (10.1016/s0378-5955(98)00078-1) 9714576

[101] Capshaw G , Diebold CA , Adams DM , Raynor J , Wilkinson GW , Moss CF , Lauer AM . 2024 Data supporting: Resistance to age-related hearing loss in the echolocating big brown bat (Eptesicus fuscus). Figshare. (10.6084/m9.figshare.26130436) PMC11708781 39500378

[102] Capshaw G , Diebold C , Adams DM , Rayner J , Wilkinson GS , Moss CF . 2024 Data from: Resistance to age-related hearing loss in the echolocating big brown bat (Eptesicus fuscus). Figshare. (10.6084/m9.figshare.c.7499986) PMC11708781 39500378

